# Correlates of consistent condom use among recently initiated and traditionally circumcised men in the rural areas of the Eastern Cape Province, South Africa

**DOI:** 10.1186/1471-2458-14-668

**Published:** 2014-06-30

**Authors:** Anam Nyembezi, Robert AC Ruiter, Bart van den Borne, Sibusiso Sifunda, Itumeleng Funani, Priscilla Reddy

**Affiliations:** 1 Population Health, Health Systems and Innovation, Human Sciences Research Council, Private Bag X9182, Cape Town 8000, South Africa; 2 Department of Work & Social Psychology, Maastricht University, P.O. Box 616, 6200 MD Maastricht, The Netherlands; 3 Department of Health Education & Health Promotion, Maastricht University, P.O. Box 616, 6200 MD Maastricht, The Netherlands; 4 HIV/AIDS, STIs and TB, Human Sciences Research Council, Private Bag X9182, Cape Town 8000, South Africa; 5 Medical Research Council, Health Promotion Research & Development Unit, PO Box 19070, Tygerberg 7505, South Africa

**Keywords:** Consistent condom use, Traditional male initiation and circumcision, STI/HIV intervention, Rural areas, Eastern Cape Province of South Africa

## Abstract

**Background:**

Consistent use of condoms is the most effective method of preventing STIs including HIV. However, recent evidence suggests that limited knowledge about HIV prevention benefits from male circumcision leads to inconsistent condom use among traditionally circumcised men. The aim of this paper is to report on the prevalence of consistent condom use and identify its psychosocial correlates to inform future HIV prevention strategies among traditionally circumcised men in rural areas of the Eastern Cape Province of South Africa.

**Methods:**

A cross-sectional study using interviewer administered fully structured questionnaires was conducted among 1656 men who had undergone initiation and traditional male circumcision in rural areas of the Eastern Cape Province of South Africa. Logistic regression was used to evaluate univariate and multivariate relationships of psychosocial correlates with consistent condom use.

**Results:**

The mean age of the participants was 21.4 years. About 45% belonged to the amaXhosa ethnic group, followed by 15.1% of the amaMpondo, 11.6% of the amaHlubi, and 27.9% from other ethnic groups. A total of 72.3% reported having a main sexual partner and of those 44.8% indicated having other sexual partners as well. About 49% reported consistent condom use and 80% used free government issued condoms, varies among ethnic groups. A total of 35.1% indicated having tested for HIV. Of those who tested for HIV, 46% reported inconsistent condom use when having sex with their sexual partners. Univariate and multivariate analyses showed a positive association between consistent condom use and the general knowledge of condom use, attitude towards condom use with main and casual sexual partners, subjective norm towards condom use with the main sexual partner, perceived self-efficacy towards condom use, positive self-esteem, beliefs about traditional male circumcision and STI protection, attitude towards gender based violence, and cultural alienation.

**Conclusions:**

The study findings reveal important target points for future cultural sensitive health education aimed at increasing consistent condom use among initiated and traditionally circumcised men in the Eastern Cape Province.

## Background

South Africa, just like many other countries in Sub-Saharan Africa faces challenges in reducing the incidence of new HIV infections. In 2010, approximately 5.5 million South Africans reportedly lived with HIV, with prevalence of about 21.8% among pregnant women receiving antenatal care aged between 15 – 24 years old in the Eastern Cape Province [1]. The ABC strategy, which includes abstinence/delay of sexual debut, being faithful to one sexual partner/serial monogamy and correct and consistent condom use with all partners, remains the standard global recommendation for STI/HIV prevention programs [2].

Condom use is promoted by the South African government and non-government organisations through intensive mass media campaigns and free distribution of condoms [3,4]. In parallel, there has been a remarkable increase in the number of young people reporting using condoms at last sexual encounter [5]. However, serious concerns remain whether young people use condoms consistently when having sexual intercourse. A recent South African nationally representative study conducted among youth in grade 8 to 11 found that only 27.1% of the learners in the Eastern Cape Province reported that they always used a condom during sex [6].

The aim of this paper is to report the prevalence of consistent condom use and its associated factors among recently initiated and traditionally circumcised men in rural areas of the Eastern Cape Province of South Africa. Initiation and traditional male circumcision (ITMC) has long been practiced as a rite of passage that marks the transition from boyhood to manhood among the Nguni and seSotho speaking ethnic groups in the Eastern Cape Province. This cultural practice falls under the legal jurisdiction of the traditional leadership in the rural areas. Traditional leaders are custodians of customs and cultural traditions practiced by their people, including ITMC, which is a cultural practice that has evolved over centuries. According to the Traditional Leadership and Governance Framework Act, 2003 (Act 41 of 2003), “traditional leader” means any person who, in terms of customary law of the traditional community concerned, holds a traditional leadership position, and is recognized in terms of this Act [7]. Traditional leaders are tasked to oversee traditional matters in the respective communities, including the safe conduct of ITMC by governing over its practices, processes, and human resources [7-9]. Adolescent boys that belong to the Nguni and seSotho ethnic groups are expected to voluntarily seek permission from their parents or family members to undergo ITMC, or otherwise be advised by parents or family to do so. In the June 2005 initiation season, a total of 10 609 boys were initiated in the Eastern Cape Province [10]. The age in recent years for participating in ITMC ranges of 16 to 26 years, with an average age of 18 years [11].

ITMC is basically the permanent removal of the foreskin by a traditional surgeon (*ingcibi*) using a traditional cutting tool (*umkhonto)* without any anesthesia. When the boy is circumcised, he becomes an initiate (*umkhwetha*) for the duration of his stay of about 28 days at the initiation lodge (*ibhoma*). His wound is nursed by a traditional guardian (*ikhankatha*) using traditional medicines. An *ikhankatha* is an initiated and traditionally circumcised man from the community who has undergone experiential learning on the care and education of the initiates. The father or family members of the *umkhwetha* appoint the *ikhankatha*. Additionally, the village men, who have also undergone the same initiation processes, visit the *ibhoma* to monitor the healing progress of the wound, teach and prepare *umkhwetha* for new roles and essential responsibilities towards his family and community members [12]. The *umkhwetha* is transformed to be a responsible man. He is also taught about appropriate sexually behaviour, such as dangers of promiscuity and about marriage and starting of a family [10,13]. The whole ITMC procedure takes about four to eight weeks to complete and is usually performed during summer and winter seasons and coincides with school holidays (e.g., in June and December). Initiated men are an important cohort to study in the context of STI/HIV prevention because of possible incorrect risk beliefs regarding the chances of HIV infection. A pilot study among recently traditional circumcised men in the Eastern Cape Province of South Africa reported that 92% of the participants believed that circumcised men do not need to use condoms during sexual intercourse [14]. A study among traditionally circumcised men in Cape Town reported that those men who were aware of the HIV preventive benefits of male circumcision and held positive beliefs about the reduced risk of HIV infection as a result of male circumcision were more likely to engage in risky sex; specifically unprotected sex and sex with multiple sexual partners [15]. In addition, the ITMC process itself may put initiates at risk for blood-borne infections by the absence of protective gloves, use of unsterile instruments, and sharing of contaminated instruments among boys in the same group [11,16,17].

As South Africa gears up to consolidate gains in HIV prevention, it is vitally important that initiated and traditionally circumcised men in the rural areas are fully involved in HIV prevention. Furthermore, women may also face the challenge of negotiating safe sex with traditionally circumcised men who think they do not have to use condoms. Therefore, understanding the factors that are associated with consistent condom use among young people particularly those who have undergone ITMC could provide crucial information for designing appropriate health education interventions aimed at increasing consistent condom use.

In the last three decades, a number of social cognitive theories have been developed to understand the determinants of health behaviours, among which the Theory of Planned Behaviour [18], Social Cognitive Theory [19], and Protection Motivation Theory [20] are the most commonly used frameworks to explain health behaviour (for integrative approaches, see [21,22]. As conceptual framework for the present study we used the Theory of Planned Behaviour [18], which has been shown to predict consistent condom use and a wide range of other behaviours [23-29]. The Theory of Planned Behaviour (TPB) proposes that intention is predicted by attitudes, subjective norms and perceived behavioural control. Attitude refers to a person’s overall evaluation of the behaviour (e.g. consistent condom use) likely to be good or bad. Subjective norm refers to person’s beliefs concerning significant other’s approval or disapproval of the behaviour. Finally, perceived behavioural control (PBC) refers to a person’s perception of whether or not they have control over behaviour, even in circumstances that would make performance of the behavior difficult. PBC is closely related to Bandura’s concept of self-efficacy, which is the estimation of one’s ability to perform the particular behaviour. We added the concept of self-efficacy as it has been found in previous studies to predictor consistent condom use [30-32]. Based on the TPB, the aim of this study was to predict the intention to consistent condom use and associated factors among recently initiated and traditionally circumcised men in rural areas of the Eastern Cape Province of South Africa. According to the TPB, attitudes, subjective norms and perceived behavioural control would predict intention to use condom consistently. In this study, measures of attitude referred to men’s perceptions regarding the advantages and disadvantages of using condoms. Subject norm referred to men’s perceived social pressure (sexual partner, men, family and community member) to use or not use a condom. Perceived behavioural control measures referred to men’s perception of whether or not they are confident to use a condom, and whether or not they can control personal condom use. We hypothesized that within the constructs of the TPB, attitude would be affected by knowledge of condoms and perceived control, that perceived opinions of community, family, men, peers and sexual partner would play an important role in explaining the intention to use condoms consistently, and that barriers such as costs of condoms would limit access to condoms.

Furthermore, to explore the influence of the cultural context in explaining consistent condom use, guided by TPB, we included measures related to the cultural and traditional belief system of ITMC as a part of a rite of passage from boyhood to adulthood in rural areas of the Eastern Cape Province of South Africa, that also contributes to the reinforcement of ethnic identity. Traditionally circumcised men are prepared for new roles as adults and are taught about essential family and community responsibilities. Therefore, it was prudent to measure beliefs about ITMC, attitude and subjective norm towards being a responsible man, perceived self-efficacy to uphold traditional values, self-esteem and ethnic identity. Ethnic identity has been described as the degree to which a person identifies with and is involved socially, politically, emotionally, behaviorally or spiritually in cultural beliefs and practices of one’s racial/ethnic group. The development of an ethnic identity, regardless of one’s ethnicity, is an essential human need because it provides the individual with a sense of belonging and historical continuity based on a common cultural heritage [33,34].

## Method

### Study design, area and participants

This cross-sectional study was conducted in five rural district municipalities of the Eastern Cape Province of South Africa. The municipalities Alfred Nzo, Amathole, Chris Hani, Joe Gqabi and OR Tambo were purposively selected because they encompass the diverse ethnic groups of people living in the Eastern Cape. Within the selected municipalities, there are 197 local traditional leaders and 90 were sampled with the assistance of the Eastern Cape House of Traditional Leaders. While all the ethnic groups in the province were sampled, some ethnic groups have more population compared to others. Therefore, we sampled more local traditional leaders from those less populated ethnic groups to ensure that all groups were represented. Within the communities of the sampled traditional leaders, participants were conveniently sampled and eligible to participate if they were isiXhosa or seSotho speaking and had undergone ITMC in the previous 12 to 24 months. Informed consent was obtained from 2337 men who then filled in an interviewer administered fully structured questionnaire. A total of 681 participants were excluded from the data analysis as they reported never having had sex. This resulted in a total sample of 1656 participants. The average duration of time between initiation and interviews was one and a half years.

### Procedure

Data were collected from January to May 2010 by forty extensively trained community research assistants (CRAs) who were identified and recruited through the local traditional leader. All CRAs were males, who had undergone ITMC, spoke either isiXhosa or seSotho as their first language, and lived in the same community as the participants. The use of an independent group of interviewers, who had no experience with ITMC and were not from the research area, would not have been accepted by the participants because of the sensitive issues surrounding ITMC and sexual behaviour. Through their extensive knowledge of the area and work experience with the local traditional leader, the CRAs were expected to be able to approach and recruit research participants during the cultural events that were related to ITMC, and obtain written consent. Selected CRAs were intensively trained for the tasks by the research team. They were familiarised with the objectives of the study, and taught how to recruit participants, obtain informed consent, to administer the questionnaire, and how to record responses on the answering sheet.

All interviews were done in isiXhosa or seSotho, the first language of both the participants and the interviewers. The interviews took place in the home of the participants or a place where they were comfortable. Detailed information about the content, procedures and confidentiality were provided verbally and in writing before written consent was obtained. Data were collected in three phases to ensure that the 40 CRAs are properly supervised by the first author to avoid inter-observer bias. In the first phase, the CRAs conducted five interviews in the presence and supervision of the first author. In the second phase, CRAs submitted ten completed questionnaires with consent forms to the first author who checked the quality, accuracy and completeness and provided feedback. In the last phase, the first author checked all the consent forms and questionnaires submitted by the CRAs and monitored the quality of the process. Ethical approval for the study was obtained from the South African Medical Association Ethics Committee (SAMAREC).

### Measures and scale construction

The development of the questionnaire was based on a review of the literature and theoretical constructs. The primary questionnaire was developed in isiXhosa to ensure face validity, and then translated into English and seSotho. All questions were inspected for cultural relevance and appropriateness by convening an instrument development workshop with members of the Eastern Cape House of Traditional Leaders, guardians and custodians who had experience with the initiation process. The dialogue focused on the content of the questionnaire, such as topics for enquiry, and how to phrase questions in a culturally sensitive manner that would not alienate the research participants or encourage socially desirable response biases. Finally, the questionnaire was pre-tested among a group of 114 recently initiated and circumcised young men living in rural areas of the Eastern Cape Province of South Africa who had similar life experiences to the research participants.

#### Socio-demographic measures

Questions using appropriate scales elicited age, ethnic group, whether they were currently in school, highest grade passed (1 = *Primary,* 2 = *Secondary,* 3 = *Post matric*), employment status, who they live with, who supports them financially, year of ITMC, and if they voluntary decided to undergo ITMC. In order to have an indication whether participants followed local and accepted procedures with regards to the initiation processes, participants were also asked if they know the name of the local traditional leader, and if they registered with the traditional leader before undergoing the initiation processes.

#### Past sexual behaviour

Sexual behaviour questions enquired about whether participants had a main sexual partner, other sexual partners, ever had sex, ever had STI, and ever tested for HIV using a scale from 0 to 1 (0 = *No*, 1 = *Yes*). Open-ended questions asked about the number of sexual partners, and the number of women one had ever had sex with in the past 6 months and past 30 days. Types of condoms used regularly was also measured using a binary scale of 1 to 2 (1 = *Government free issued condoms*, 2 = *Bought condoms*).

#### Consistent condom use

The main outcome variable of interest was consistent condom use behaviour, which was measured using the averaged scores of three items: “When you have sex, how often do you use a condom” (1 = *Never*, 2 = *Sometimes*, 3 = *Always*), “The last time you had sex did you use a condom” (0 = *No*, 1 = *Yes*), and “Did you use a condom when you had sex for the first time after initiation processes” (0 = *No*, 1 = *Yes*). We decided to dichotomize the scores such that meaningful binary answering categories were created by differentiating those that scored positively on the variable of interest from those that gave a neutral or negative response. Participants obtained “consistent condom use” (1 = *Yes*, 0 = *No*) if they reported to have always used condoms, used it at last sex and after ITMC (49.2%). If participants failed to score positive on at least one of the items they were denoted as inconsistent condom users (44.9%).

#### Psychosocial measures

All social psychological measures were based on Likert-type items with five response options unless otherwise indicated. For each measure, scores on items that showed sufficient internal consistency (Cronbach’s alpha [α] > .60) were averaged into one single index. Higher scores reflect a stronger presence of the concerning variable.

#### Condoms

*Knowledge of protective effects of condoms* was assessed with three items (e.g., ‘If used properly and regularly, condoms can prevent STIs’; α = .88), using a scale with three possible options (true, false, I do not know). Those who had one or more wrong answers were coded 0 = incorrect (13.5%), whereas those who scored all three items correctly were coded as 1 = correct (85.1%). The same procedure was used to assess knowledge of appropriate use of condoms using four items (e.g., ‘Male condoms can break if the tip is not pinched to remove air bubbles’; α = .66). Those who had one or more wrong answers were coded as 0 = incorrect (73.5%), whereas those who scored all four items correctly were coded as 1 = correct (22.0%). Thirteen semantic differentials were used to measure participant’s *attitude towards condom use with a casual sexual partner* (e.g., ‘I feel valuable – I feel worthless’, ‘I feel good – I feel bad’; α = .95). In a similar way, *attitude towards condom use with the main sexual partner* were assessed by the mean scores of thirteen semantic differentials (e.g., ‘I feel safe – I feel risky’, ‘I feel trustworthy – I feel untrustworthy’; α = .96). *Subjective norm towards condom use with casual partners* were assessed by the means scores of five items (e.g., ‘Most of community members think that you should use a condom when you have sex with a casual sexual partner’; α = .77), using a five-point scale (1 = strongly disagree, 5 = strongly agree). *Subjective norm towards condom use with the main sexual partner* was assessed by the means scores of six items (e.g., ‘Most of family members think that you should use a condom when you have sex with your main sexual partner’; α = .90). *Self-efficacy towards condom use* was assessed by the means scores of five items (e.g., ‘How confident are you that you will be able to use a condom correctly the next time you have sex?’; α = .61), using a five-point scale (1 = not confident at all, 5 = very confident). *Perceived personal barriers on condom access* were assessed by the mean score of three items (e.g., ‘I am scared to ask for condoms’; α = .89). *Perceived organisational barriers on condom access* were assessed by the mean score of three items (e.g., ‘I have to travel far to access condoms’; α = .71).

#### Self-esteem

Self-esteem was measured by 10 items with a five-point scale. Explorative factor analysis using principal axis factoring as extraction method was conducted. This analyses revealed that the scores on the 10 items converged to a two-factor solutions with a group of items focusing on positive aspects of the self (positive self-esteem), and a group of items focusing on negative aspects of the self (negative self-esteem). *Positive self-esteem* was assessed by the means scores of six items (e.g., ‘On the whole I feel good about myself’; α = .80). *Negative self-esteem* was assessed by the means scores of four items (e.g., ‘I feel I am a failure’; α = .80).

#### Violence

*Attitude towards gender-based violence* were assessed by the mean score of seven items (e.g., ‘A man’s got to show the woman who’s boss right from the start or he’ll end up ‘hen-pecked’; α = .79). *Attitude towards sexual coercion* were assessed by the mean score of four items (e.g., ‘Sometimes the only way a man can get a woman turned on is to use force’; α = .68). *Subjective norm towards gender based violence* were assessed by the means scores of three items (e.g., ‘Most of your community members think that a responsible man is someone who has to discipline his wife/partner when necessary using force’; α = .96).

#### Responsible manhood

*Attitude towards being a responsible man* were measured with two items (e.g., ‘A responsible man is someone who looks after his partner’s wellbeing’; *r* = .39). *Subjective norm towards responsible man’s positive support* were assessed by the means scores of three items (e.g., ‘Most of your community members think that a responsible man is someone who supports his partner and children both financially and emotionally’; α = .83). *Subjective norm towards responsible man’s family welfare* were assessed by the means scores of three items (e.g., ‘Most of your community members think that a responsible man is someone who looks after his partner’s wellbeing’; α = .82). *Subjective norm towards traditionally circumcised men* were assessed by the means scores of six items (e.g., ‘Most people in the community expect traditionally circumcised men to behave in a respectful manner’; α = .82). *Perceived self-efficacy to uphold traditional values* was assessed by the means scores of three items (e.g., ‘How confident do you feel that as a man you would do what is expected from you?’; α = .79). *Perceived self-efficacy of being a responsible man* was assessed by the means scores of four items (e.g., ‘How confident are you that you will be able to look after your partner’s wellbeing?’; α = .80). *Beliefs about male circumcision and STI protection* were assessed by the means scores of four items (e.g., ‘Male circumcision helps to prevent chances of men contracting HIV’; α = .90). *Beliefs about traditional male circumcision as a positive experience* were assessed by the means scores of four items (e.g., ‘My belief is that undergoing traditional male circumcision is important’; α = .81). *Received general teachings about being a responsible man* were assessed by the means scores of twenty-six items (e.g., ‘In the traditional male initiation school I was taught about respect’; α = .96). *Received teachings about traditional related practices* were assessed by the means scores of three items (e.g., ‘In the traditional male initiation school I was taught about the effectiveness of traditional medicines for wound healing’; α = .81). *Received teachings about sexual behaviour aspects* were assessed by the means scores of three items (e.g., ‘In the traditional male initiation school I was taught not to have multiple sexual partners’; α = .83).

#### Ethnic identity

Ethnic identity was assessed using 17 items adapted from the Adult Survey of Black Life and Adolescent Survey of Black Life [35,36], with a 5-point Likert scale (1 = strongly agree, 5 = strongly disagree). Factor analysis suggested two subscales which were coded as cultural affiliation and cultural alienation. The subscale of *cultural affiliation* was measured by nine items (e.g., ‘I am proud of my cultural heritage’; α = .87). The subscale of *cultural alienation* was measured by seven items (e.g., ‘There is still a lot of racism in this country’; α = .89). All items were coded so that higher values indicated more cultural affiliation or more cultural alienation.

### Data analysis

Frequencies and percentages, or means and standard deviations (SD), were used to describe categorical and continuous variables, respectively. Because of the highly negative skewed distributions on most psychosocial measures (i.e. participants scoring very positive), we decided to dichotomise psychosocial variables concepts, unless otherwise indicated. Logistic regression was used to evaluate univariate relationships with consistent condom use. The variables displaying significant values of p < 0.05 in the univariate analysis were included in the multivariate regression model. Estimations of odds ratio (OR), 95% confidence interval, (CI), and p values are presented for all analyses. All analyses were conducted in SPSS Version 16 (SPSS Inc., Chicago, IL).

## Results

The results of the analysis are organized in five sub-sections and two tables. Below we summarize the main findings.

### Socio-demographics

The mean age of participants was 21.4 years (SD = 2.22). The majority of the participants (45.4%) identified themselves as belonging to the amaXhosa ethnic group, followed by 15.1% of the amaMpondo, and then 11.6% of the amaHlubi. The other 27.9% of the participants were from other ethnic groups among which were abaThembu, amaZizi, abeSotho, amaBhaca, amaMpondomise, amaBhele and amaXesibe. Almost 63% of the participants reported living with their parents, 92.9% were financially supported by their parents and family, 38.3% were currently in school, 13.9% were currently working, and the remainder were unemployed. Of the participants, 91.1% were able to identify their local traditional leader by name. A total of 66.5% reported that they got permission and registered with their traditional leader prior to the proceedings of ITMC processes. The majority of the participants (82.1%) voluntary decided to undergo ITMC. About 55% of men were traditionally circumcised in 2007, whilst 40.8% and 2.6% were circumcised in 2008 and 2009 respectively Table 1.

**Table 1:** Demographic and sexual profile of participants (N = 1656)

**Variables**	**Frequencies**	**Percentage**
**Ethnic groups**		
AbaThembu	36	2.2%
AmaMpondo	250	15.1%
AmaHlubi	192	11.6%
AmaZizi	36	2.2%
AmaXhosa	751	45.4%
AbeSotho	64	3.9%
AmaBhaca	68	4.1%
AmaMpondomise	79	4.8%
AmaBhele	62	3.7%
AmaXesibe	47	2.8%
Other	71	4.3%
**Living**		
Alone	36	2.2%
Parents	1038	62.7%
Family	472	28.5%
Other	110	6.6%
**Financial support**		
Yourself	80	4.8%
Parents and family	1539	92.9%
Friends	20	1.3%
Other	17	1.0%
**Currently in school**		
Yes	634	38.3%
No	1022	61.7%
**Highest grade passed**		
Primary	349	21.1%
Secondary	1047	63.2%
Post-matric	260	15.7%
**Currently working**		
Yes	230	13.9%
No	1426	86.1%
**Have children**		
Yes	260	15.7%
No	1396	84.3%
**Know your traditional leader**		
Yes	1508	91.0%
No	148	9.0%
**Registered with the traditional leader before initiation**		
Yes	1101	66.5%
No	555	33.5%
**Voluntary undergone initiation**		
Yes	1377	83.2%
No	279	16.8%
**Year of circumcision**		
2007	877	55.4%
2008	647	40.8%
2009	41	2.6%
**Main sexual partner**		
Yes	1198	72.3%
No	456	27.5%
**Other sexual partner**		
Yes	523	44.8%
No	644	55.2%
**Reported STI**		
Yes	196	11.8%
No	1390	83.9%
**Consistent condom use**		
Always	923	55.7%
Sometimes	499	30.1%
Never	181	10.9%
**Condom use at last sex**		
Yes	1232	74.4%
No	317	19.1%
**Condom use after initiation**		
Yes	1180	71.3%
No	409	24.7%
**Frequently used condom**		
Government free issued	1323	79.9%
Bought	177	10.7%
**HIV test**		
Yes	581	35.1%
No	1033	62.4%

### Sexual behaviours

A total of 72.3% participants in the study reported having a main sexual partner and of those 44.8% indicated having other sexual partners as well. The mean number of other sexual partners was 2.21 (SD = 3.23). The mean number of female sexual partners in the past 6 months and past 30 days was 1.63 (SD = 1.27) and 1.32 (SD = 1.13) respectively. A total of 11.8% reported having previously contracted a sexually transmitted infection. In total, 815 (49.2%) of the participants reported consistent condom use when having sex, varies among ethnic groups. The majority (79.9%) reported that they regularly use government free issued condoms. A total of 35.1% indicated having tested for HIV. Of those who tested for HIV, 46% reported inconsistent condom use when having sex with their sexual partners.

### Beliefs about male circumcision and STI protection

Of the participants in the study, 54.4% agreed with the belief that male circumcision helps to prevent the chances of men contracting STIs. A total of 54.7% agreed with the belief that male circumcision helps to prevent the chances of men contracting HIV. The belief that male circumcision helps to prevent the spread of STIs to women was reported by 47% of participants. A total of 48.6% believed that male circumcision helps to prevent the spread of HIV to women.

### Univariate model of consistent condom use

The results of univariate tests of independence between consistent condom use and psychosocial variables are presented in Table 2. Consistent condom use was significantly associated with knowledge of protective effects of condoms, knowledge of appropriate use of condoms, attitude towards condom use with a casual sexual partner, attitude towards condom use with the main sexual partner, subjective norm towards condom use with the main sexual partner, self-efficacy towards condom use, received teachings about sexual behaviour aspects, and beliefs about male circumcision and STI protection.

**Table 2:** Results of univariate and multivariate logistic models with consistent condom use as dependent variable

**Variables**	**Participants consistent condom use**		**Univariate model**		**Multivariate model**	
	**No (N = 744)**	**Yes (N = 815)**	**OR (95% CI)**	**P-value**	**OR (95% CI)**	**P-value**
Knowledge of protective effects of condoms	81%	92%	1.64 (1.37, 1.97)	<.001	1.32 (1.08, 1.62)	<.001
Knowledge of appropriate use of condoms (Mean, SD)	2.23 (1.28)	2.70 (1.17)	1.36 (1.25, 1.48)	<.001	1.21 (1.09, 1.34)	<.001
Attitude towards condom use with a casual sexual partner	65%	84%	1.48 (1.34, 1.64)	<.001	1.16 (1.02, 1.32)	<.05
Attitude towards condom use with the main sexual partner	33%	57%	1.39 (1.29, 1.49)	<.001	1.14 (1.04, 1.27)	<.001
Subjective norm towards condom use with a casual sexual partner	70%	73%	1.07 (0.92, 1.24)	NS	-	-
Subjective norm towards condom use with the main sexual partner	31%	49%	1.49 (1.35, 1.64)	<.001	1.19 (1.03, 1.37)	<.01
Self-efficacy towards condom use	53%	70%	2.04 (1.77, 2.35)	<.001	1.78 (1.46, 2.17)	<.001
Perceived personal barriers on condom access	52%	35%	0.73 (0.67, 0.79)	<.001	0.78 (0.68, 0.89)	<.001
Perceived organizational barriers on condom access	56%	41%	0.84 (0.76, 0.94)	<.001	1.06 (0.90, 1.23)	NS
Positive self-esteem	85%	88%	0.67 (0.55, 0.81)	<.001	1.45 (1.06, 1.99)	<.01
Negative self-esteem	48%	57%	0.94 (0.85, 1.04)	NS	-	-
Received general teachings about being a responsible man	90%	78%	0.46 (0.37, 0.57)	<.001	0.42 (0.28, 0.63)	<.001
Received teachings about traditional related practices	65%	68%	0.94 (0.85, 1.03)	NS	-	-
Received teachings about sexual behaviour aspects	47%	53%	1.20 (1.10, 1.31)	<.001	1.10 (0.98, 1.23)	NS
Beliefs about male circumcision and STI protection	39%	46%	1.17 (1.06, 1.29)	<.001	1.16 (1.02, 1.32)	≤.05
Beliefs about male circumcision as a positive experience	90%	83%	0.57 (0.48, 0.67)	<.001	0.93 (0.72, 1.21)	NS
Subjective norm towards traditionally circumcised men	79%	76%	0.71 (0.61, 0.83)	<.001	0.94 (0.76, 1.17)	NS
Perceived self-efficacy to uphold traditional values	92%	95%	1.07 (0.89, 1.28)	NS	-	-
Attitude towards being a responsible man	88%	88%	0.84 (0.71, 0.98)	<.05	0.93 (0.73, 1.18)	NS
Subjective norm towards responsible man’s positive support	79%	86%	1.08 (0.94, 1.24)	NS	-	-
Subjective norm towards responsible man’s family welfare	70%	83%	0.69 (0.60, 0.80)	<.001	0.69 (0.56, 0.85)	<.001
Subjective norm towards gender based violence	57%	59%	1.00 (0.92, 1.09)	NS	-	-
Perceived self-efficacy of being a responsible man	93%	93%	0.86 (0.71, 1.04)	NS	-	-
Attitude towards gender based violence	42%	31%	0.80 (0.71, 0.90)	<.001	1.24 (1.03, 1.48)	<.01
Attitude towards sexual coercion	23%	15%	0.57 (0.44, 0.73)	<.001	0.81 (0.70, 0.95)	<.01
Cultural affiliation	93%	88%	0.54 (0.43, 0.67)	<.001	0.45 (0.24, 0.83)	<.01
Cultural alienation	83%	74%	0.51 (0.40, 0.65)	<.001	2.07 (1.21, 3.54)	<.001

*NS* = not significant.

Furthermore, consistent condom use was negatively associated with perceived personal barriers on condom access, perceived organisational barriers on condom access, positive self-esteem (positive aspect of self), received general teachings about responsible man, beliefs about ITMC as a positive experience, subjective norm towards traditionally circumcised men (perceived expectations towards traditionally circumcised men), attitude towards being a responsible man, social norm towards responsible man’s family welfare, attitude towards gender based violence, attitude towards sexual coercion, ethnic identity towards cultural affiliation and ethnic identity towards cultural alienation. There was no univariate association between consistent condom use and subjective norm towards condom use with casual sexual partner(s), negative self-esteem (negative aspect of self), received teachings about traditional related practices, perceived self-efficacy to uphold traditional values, subjective norm towards responsible man’s positive support, subjective norm towards gender based violence and perceived self-efficacy of being a responsible man.

### Multivariate model of consistence condom use

Variables that revealed significant univariate associations with consistent condom use were further explored in a multivariate logistic model to determine whether these associations were unique and independent (see Table 2). Men who reported consistent condom use were more likely to indicate knowledge of protective effects of condoms, knowledge of appropriate use of condoms, positive attitude towards condom use with a casual sexual partner, positive attitude towards condom use with the main sexual partner, subjective norm towards condom use with the main sexual partner, perceived self efficacy towards condom use, positive self-esteem, beliefs about male circumcision and STI protection, attitude towards gender based violence, and ethnic identity towards cultural alienation. In contrast, perceived personal barriers on condom access, received general teachings about responsible man, subjective norm towards responsible man’s family welfare, attitude towards sexual coercion and ethnic identity towards cultural affiliation were inversely related to consistent condom use condoms.

## Discussion

The results of this study highlight some important aspects about sexual behaviours that put young people at risk of sexually transmitted infections. This group of young men is an important cohort to study in the context of STIs/HIV transmission and unwanted pregnancies, as they have now undergone a cultural rite of passage that makes it socially acceptable to initiate sexual relations. Interestingly, 91.1% of the participants were able to identify their traditional leaders and 65.5% registered with them prior to proceedings of ITMC. Therefore, the majority of the participants were highly likely to have followed the local procedures and legislation processes that provides for the correct and safe conduct of ITMC. However, the pre-induction sexual activity represents a shift in the sexual behaviour practices of young men who undergo ITMC processes when compared to the past, where the majority of young men underwent circumcision before engaging in sexual activities (interview with ECHOTL chairperson Nkosi Matanzima). In rural African communities there has always been a popular common practice and social expectations of abstinence from sex until marriage [37]. In communities where male circumcision is traditionally and culturally practiced, only circumcised men were prepared for sex, and could therefore have sexual relationships and marry [10].

This study expands our understanding of condom use in populations at risk by exploring correlates of consistent condom use among recently initiated and traditionally circumcised men in the rural areas of Eastern Cape Province, South Africa. Our sample is at increased risk for STIs/HIV infection because about 45% were in concurrent sexual relationships and almost half reported inconsistent condom use. Given that the mean age was 21 years old, the participants represent a group that is regarded as highly susceptible to STIs/HIV infection and spread to their female sexual partners. On a more positive note, almost 80% of the condoms that were used by the participants were provided by government. This could suggest that South African government’s free distribution of condoms is effective even in the rural areas of Eastern Cape Province, therefore its delivery should be continued. However, it is equally important to help young people to effectively use condoms by designing appropriate interventions aimed at increasing consistent condom use. Men who have higher knowledge of protective effects and appropriate use of condoms were more likely to use them consistently. Therefore, this emphasizes the importance of maintaining a high level of condom knowledge, particularly concerning consistent condom use in HIV prevention programmes targeting young people. In addition, intervention programs should provide circumcised men with the necessary skills that are designed to promote negotiation with their sexual partners to always use condoms.

In line with others studies, our findings support the idea that an extended TPB provides a good model for identification of correlates of consistent condom use [23-32]. The positive association between consistent condom use and attitude towards condom use with a casual and main sexual partner may well be explained by participants understanding of the effectiveness of condoms. When risk is perceived, it is logical to expect that people will seek ways to protect themselves and inform themselves about the effectiveness of such means.

With regards to subjective norm, significant others were reported by the participants as more likely to approve consistent condom use among initiated and traditionally circumcised men when having sex with the main sexual partner. It maybe that prevention of unwanted pregnancy influenced their support for consistent condom use among steady relationships. Somewhat counterintuitive were the findings that subjective norm towards condom use with a casual sexual partner was not significantly associated to consistent condom use. One possible explanation for this finding could be that significant others expect initiated and traditionally circumcised men to be responsible and engage in monogamous relationships, therefore were less concerned about casual sexual partners. Those participants that perceived personal barriers towards access to condom were less likely to use them consistently. These findings suggest that there is a need for prevention interventions designed to make consistent condom use more socially acceptable. In addition, the absence of condom use barriers is also very important in promoting effective HIV prevention.

Positive self-esteem and self-efficacy towards condom use were associated with the outcome variable. Those participants who were comfortable with insisting on using condoms as well as possessing the practical skills of correctly using them were more motivated to use condoms consistently than those who were less confident in performing those tasks. These findings stress the importance of perceived and actual skill to practice the behavior as well being able to communicate safer sex practice with a partner as strong predictors of engaging in behaviour.

With regards to measures related to the cultural and tradition belief system of male initiation and circumcision, beliefs about male circumcision and STI protection were positively associated with consistent condom use. However, received general teachings about responsible man and subjective norm towards responsible man’s family welfare were negatively associated to the dependent variable. This negative association may well be explained by participant’s belief that a responsible man does not need to use a condom consistently as he ought to be faithful to his sexual partner. Tailored interventions with an objective to increase consistent condom use among initiated and traditionally circumcised rural men in the Eastern Cape Province could consider including components aimed to discourage any attitude that promote gender based violence and sexual coercion.

The current investigation has several limitations that should be considered. The interpretation of the results is limited to the population in which the research was done, and further research should be extended to traditionally circumcised men of different populations, cultures and settings. For example, further researcher could possibly be done among Ndebele and Pedi populations of South Africa. The community research assistants who conducted the interviews were from the same community as the participants. Therefore, they knew each other and it is possible that some of the participants gave bias responses. Another limitation is the use of self-reported measures to assess sensitive and complex information about condom use, which leaves room for misreporting and socially approved answers. These limitations notwithstanding, this study generated new measures and constructs on this topic as well as the reliability of these measures.

## Conclusion

The results of this study highlight the high prevalence of risky sexual behaviours that put young men and their sexual partners at risk of STIs/HIV and unwanted pregnancies, which seem to be influenced by a wide spectrum of psychosocial factors. The predictors identified by this study reveal opportunities for the development of focused culturally sensitive health education aimed to increase consistent condom use among initiated and traditionally circumcised men in the Eastern Cape Province. Health education interventions could consider including components aimed to increase men’s knowledge of protective effects and appropriate use of condoms, increase self-efficacy towards condom use, and strengthen positive attitude and perceived subjective norm towards condom use, which can be integrated into the initiation and traditional teachings about sexual behaviours.

## Competing interests

The authors declare that they have no competing interests.

## Author’s contributions

AN – was involved in research instrument development, data collection, conceptualization of the paper, data analysis and wrote the paper. RACR – was involved in conceptualization of the paper, data analysis and writing of the paper. BVDB – was involved in conceptualization of the paper, data analysis and writing of the paper. SS – was involved in research instrument development, conceptualization of the paper, data analysis and writing of the paper. IF – was involved in research instrument development, data collection, conceptualization of the paper, data analysis and wrote the paper. PR – was involved in research instrument development, conceptualization of the paper, data analysis and wrote the paper. All authors read and approved the final manuscript.

## Authors’ information

Co-authors: Robert A.C. Ruiter; Bart van den Borne; Sibusiso Sifunda; Itumeleng Funani & Priscilla Reddy.

## Pre-publication history

The pre-publication history for this paper can be accessed here:

http://www.biomedcentral.com/1471-2458/14/668/prepub
